# Bioassay-Guided Isolation of Guggulsterol I and Evaluation of Its Antioxidant Properties in Ethanol Extracts From Commiphora caudata Leaves

**DOI:** 10.7759/cureus.68166

**Published:** 2024-08-29

**Authors:** Karupanagounder Thangaraj Uthra, Krishnan Manikandan, Thirumal Margesan, Gururaja Perumal Pazhani

**Affiliations:** 1 Department of Pharmaceutical Chemistry, Sri Ramaswamy Memorial (SRM) College of Pharmacy, SRM Institute of Science and Technology, Chengalpattu, IND; 2 Department of Pharmaceutical Analysis, Sri Ramaswamy Memorial (SRM) College of Pharmacy, SRM Institute of Science and Technology, Chengalpattu, IND; 3 Department of Pharmacognosy, Sri Ramaswamy Memorial (SRM) College of Pharmacy, SRM Institute of Science and Technology, Chengalpattu, IND

**Keywords:** hptlc, column chromatography, guggulsterol i, antioxidant activity, commiphora caudata

## Abstract

The secondary metabolites of various parts of *Commiphora caudata* have shown a range of biological activities under both in vivo and in vitro conditions, particularly anti-inflammatory and antioxidant properties. While E-guggulsterone from this plant has been proven to have anti-inflammatory effects, the antioxidant potential of phytochemicals present in the leaves of *C. caudata* is less explored. This investigation aimed to isolate an antioxidant phytoconstituent from the ethanolic extract of the dried leaves of *C. caudata* using a bioassay-guided approach. The dried leaves were successively extracted with organic solvents, including ethanol. The presence of phytochemicals was tested using high-performance thin-layer chromatography (HPTLC), and phytoconstituent from ethanol extract was purified by column chromatography. The antioxidant activity of both the crude extract and the purified compound was evaluated and then compared. The radical scavenging activity was measured using the 2,2-diphenyl-1-picrylhydrazyl (DPPH) assay. The ethanolic extract of *C. caudata* showed 87.6% DPPH radical scavenging activity at a concentration of 250 µg/mL, while standard ascorbic acid showed 94.9% inhibition at the same concentration. The concentrated ethanolic extract of *C. caudata* was subjected to silica-based column chromatography with an ascending polarity of mobile phase solvents, ethyl acetate, and ethanol (25:75), yielding a single compound. The isolated compound was confirmed as guggulsterol I by ultraviolet (UV), infrared (IR), mass, and nuclear magnetic resonance (NMR) spectroscopy. The radical scavenging activity of the crude ethanolic extract of *C. caudata* leaves and the isolated compound guggulsterol I was concentration-dependent. The crude ethanolic extract of *C. caudata* showed significant antioxidant activity in comparison with the standard. However, the isolated guggulsterol I showed less antioxidant activity than the crude ethanolic extract. This study strongly suggests that the crude ethanolic extract of *C. caudata* leaves had better antioxidant activity due to the synergistic or additive effect of guggulsterol I and other phytoconstituents.

## Introduction

Medicinal plants are a crucial source of alternative therapy in Asia and other developing countries. Countries such as China, India, Japan, Pakistan, Sri Lanka, Indonesia, and Thailand practice traditional medicine. The Indian traditional system, known as AYUSH, is one of the oldest medical practices and is officially recognized in India [1]. AYUSH employs a holistic approach, promoting well-being through products derived from plants, animals, or minerals [2]. In China, traditional pharmaceuticals account for about 40% of total medicinal consumption [3], while in India, conventional medical systems make up less than 30% of household therapeutic use. Variations in regional usage patterns are influenced by the availability of resources, which drive the ongoing development of traditional medical systems and the search for pharmacological applications of medicinal plants [4]. Many medicinal plants are rich sources of secondary metabolites with biological activities. There is a continuous search for these plants and their active secondary metabolites responsible for pharmacological effects. For instance, *Commiphora caudata* (Wight and Arn), native to the Western Peninsula, Sri Lanka, and India, belongs to the Burseraceae family. The genus *Commiphora* includes various species that grow as shrubs and trees in Africa, Madagascar, America, and Arabia. In African countries, the roots, leaves, and stems of *Commiphora* have traditionally been used to treat a range of conditions, including stomach aches, menstrual issues, ailments associated with spirits, malaria, wound healing, cancer, ulcers, rheumatic diseases, colds, nasal congestion, and coughing [5].

This plant naturally grows in several states of India, including Gujarat, Karnataka, Madhya Pradesh, Rajasthan, and Tamil Nadu, primarily in arid, sandy, sandy loam, and rocky areas. It is known by the trade names "raw mango" and "uphill mango" due to the raw mango-like odor of its leaves and bark in southern India [6]. Several species of *Commiphora* produce gums, oils, and resins [7]. Resins from this genus are used in making incense and perfume. Experimental studies have shown that other parts of the plant possess bioactive properties, including anti-inflammatory, antimicrobial, antimalarial, hypolipidemic, hepatoprotective, and antioxidant effects [8]. Oleo-gum resins from the *Commiphora* genus contain over 300 phytochemical compounds, including steroids and mono-, di-, and sesquiterpenes [9]. Guggul, the oleo-gum resin, has been used in Ayurvedic medicine for more than 10 centuries to treat hypertension, atherosclerosis, rheumatism, and obesity [10]. Oleo-gum resins are soluble in ethyl alcohol (30%-60%) and water (20%-40%) [11]. Various species of *Commiphora* are found in India. In Tamil Nadu, however, *Commiphora berryi* and *Commiphora caudata* are considered rare, and *Commiphora wightii* is regarded as an endangered plant [12]. The literature indicates that the leaves of *C. caudata* are widely used in traditional medicine in Tamil Nadu for their anti-inflammatory and analgesic properties, which have been scientifically evaluated [13].

The extract of *C. caudata* bark, when analyzed with hexane, ethyl acetate, and methanol, revealed 52 bioactive compounds through gas chromatography/mass spectrometry (GC/MS) analysis [14]. However, the bioactive compounds present in the leaves of *C. caudata* have not yet been studied. Isolating specific bioactive compounds through bioassay-guided fractionation is a reliable method for exploring plant potential and identifying new drug leads. Therefore, this study aimed to identify the bioactive compounds in the ethanol extract of *C. caudata* leaves using bioassay-guided fractionation and to evaluate their antioxidant activity.

## Materials and methods

Plant collection and extraction

The leaves of *C. caudata* were purchased from an Ayurvedic shop in Chennai, Tamil Nadu, in January 2019 and authenticated by Prof. P. Jayaraman, Director, The Plant Anatomy Research Centre, Tambaram, Tamil Nadu, under registration number PARC/2019/3882. Air-dried, pulverized *C. caudata* leaves (200 g) were macerated four times, each with 800 mL of n-hexane for 24 hours. After filtration, the filtrate was concentrated under reduced pressure, yielding 1.37 g of residue. The residue was then extracted sequentially with n-hexane, chloroform, ethyl acetate, and ethanol [15]. The filtrate from each solvent extraction was concentrated under reduced pressure, and the resulting residues were stored in airtight containers for subsequent analysis.

Qualitative detection of phytoconstituents

The phytoconstituents of each concentrated extract were assessed for alkaloids, carbohydrates, flavonoids, saponins, tannins, phytosterols, proteins, amino acids, terpenes, and glycosides using the methods described by Trease and Evans (1989), and Evans (2009) [15], and Harborne (1998) [16].

High-performance thin-layer chromatography (HPTLC) fingerprinting of extracts

All concentrated extracts were analyzed using the HPTLC fingerprinting method described by Harborne (1998) [16]. Each extract (100 mg) was transferred to a centrifuge tube and solubilized in 1 mL of methanol by vortex and clarified by centrifugation at 3,000 rpm for 10 minutes. The clear supernatant was used for further analysis. The chromatogram was developed using a CAMAG (Muttenz, Switzerland) HPTLC system, an automatic sample applicator with a scanner. The test sample (2 μL) was spotted on a precoated silica gel 60 F254 HPTLC plate (Merck, Mumbai, India), with appropriate space from the bottom and also the side using a Hamilton syringe. After application, the plate was incubated in an iodine chamber for 10-15 minutes. The chromatogram was developed in a glass chamber (CAMAG, Switzerland) with a pre-saturated mobile phase of toluene-ethyl acetate-formic acid-methanol (6:2:1.5:0.5) to separate phytoconstituents. After development, the thin-layer chromatography (TLC) plate was incubated in an oven at 60°C for 10 minutes. Densitometric scanning was then carried out using winCATS 1.4.2 software (CAMAG, Switzerland), with an absorption spectrum range from 200 nm to 400 nm.

DPPH radical scavenging assay (RSA)

The free radical scavenging activity of the ethanolic extract of* C. caudata *leaves was assessed using 2,2′-diphenyl-1-picrylhydrazyl (DPPH) with minor modifications to an established method [17]. A 0.1 mM/L DPPH solution was freshly prepared in methanol by vortexing. Various concentrations of the ethanolic extract of *C. caudata* and column-eluted fractions (ethyl acetate and ethanol fractions) were prepared by serial dilution, ranging from 250 µg/mL to 50 µg/mL. The free radical scavenging activity was evaluated by combining 2 mL of freshly prepared DPPH solution with 2 mL of each test solution in glass tubes. The mixture was then vigorously shaken and kept in a dark place at room temperature for 15 minutes. After incubation, the absorbance was recorded at 517 nm using a UV spectrophotometer. The radical scavenging activity was calculated from the absorbance values. Ascorbic acid and ethanol were used as controls in each assay batch. The formula for the percentage of antioxidant activity is as follows: percentage of antioxidant activity = ((Abc−Abt) ÷Ac) × 100, where Abc is absorbance (control) and Abt is absorbance (test).

Separation of phytoconstituents by column chromatography

A cylindrical glass column (500 mm in length) was packed with 80 g of silica gel (60-120 mesh) slurry made with n-hexane. The slurry was poured into the column and allowed to settle by gravity, with cotton wool used to trap any leakage of silica gel during elution. The column was stabilized by eluting it with 500 mL of n-hexane. The concentrated ethanol extract (1.4 g) was mixed with silica gel to form a fine powder, which was then added to the pre-packed column and covered with a layer of cotton. Solvents such as chloroform, ethyl acetate, and ethanol were used in ratios of 100, 75:25, 50:50, and 25:75, corresponding to different polarities. These solvents were eluted through the column at uniform rates by gravity to fractionate the test extract. Five fractions were collected separately for each solvent combination in tubes and numbered consecutively for further analysis using thin-layer chromatography (TLC). Each eluted fraction was spotted on TLC plates using a capillary tube and eluted with a mobile phase of ethyl acetate-methanol-water (20:12:8). Then, the plates were dried at room temperature. The developed TLC plates were incubated in an iodine chamber and visualized under UV light at 254 nm. Compound spots were marked, and retention factor (Rf) values were calculated.

Spectroscopy analysis

The ultraviolet spectrum of the isolated compound was obtained using a Jasco V-730 spectrophotometer (Tokyo, Japan). For mass analysis, the test sample (1 μL) was mixed with matrix solution (1 μL) spotted onto a steel target plate as per manufacture directions and dried at room temperature. The sample was analyzed using a Bruker UltrafleXtreme matrix-assisted laser desorption/ionization-time of flight (MALDI-TOF) mass spectrometer at the SRM Institute of Science and Technology, Biotechnology Facility, Tamil Nadu, India. The spectrum was obtained with the following settings: the ion source voltage was adjusted to 20 kV, and the lens voltage was set to 8.05 kV. The laser intensity varied between 55% and 70%, while the mass range spanned from 100 to 1,500 Da. A single mass spectrum was collected from every spot, with a peak width tolerance of 0.2 m/z. The average spectrum was then trimmed to include only the range from 100 to 1,500 m/z.

Fourier transform infrared (FTIR) analysis was used to characterize the functional groups in the column-eluted compound using KBr pellets on a Bruker Alpha II spectrometer (Bruker Corporation, Ettlingen, Germany). The sample was scanned in the range of 4,000-500 cm⁻¹, and the percentage transmittance versus wavenumber was recorded. The 1H NMR spectra were recorded using a JEOL ECS 400 MHz instrument with CDCl₃, and chemical shifts (δ) were reported in ppm.

## Results

In the present study, leaves were defatted with n-hexane and successively extracted with chloroform, ethyl acetate, and ethanol. The yields were 1.37 g (0.6%), 0.57 g (0.2%), 0.94 g(0.47%), and 1.62 g (0.81%), respectively. The hexane extract was found to contain steroids, glycosides, and terpenoids. The chloroform extract tested positive for alkaloids, amino acids, flavonoids, glycosides, and steroids. Ethyl acetate extract tested positive for glycosides, steroids, and flavonoids. The ethanol extract contained carbohydrates, amino acids, gum resin, tannins, glycosides, steroids, and terpenoids.

**Figure 1:**
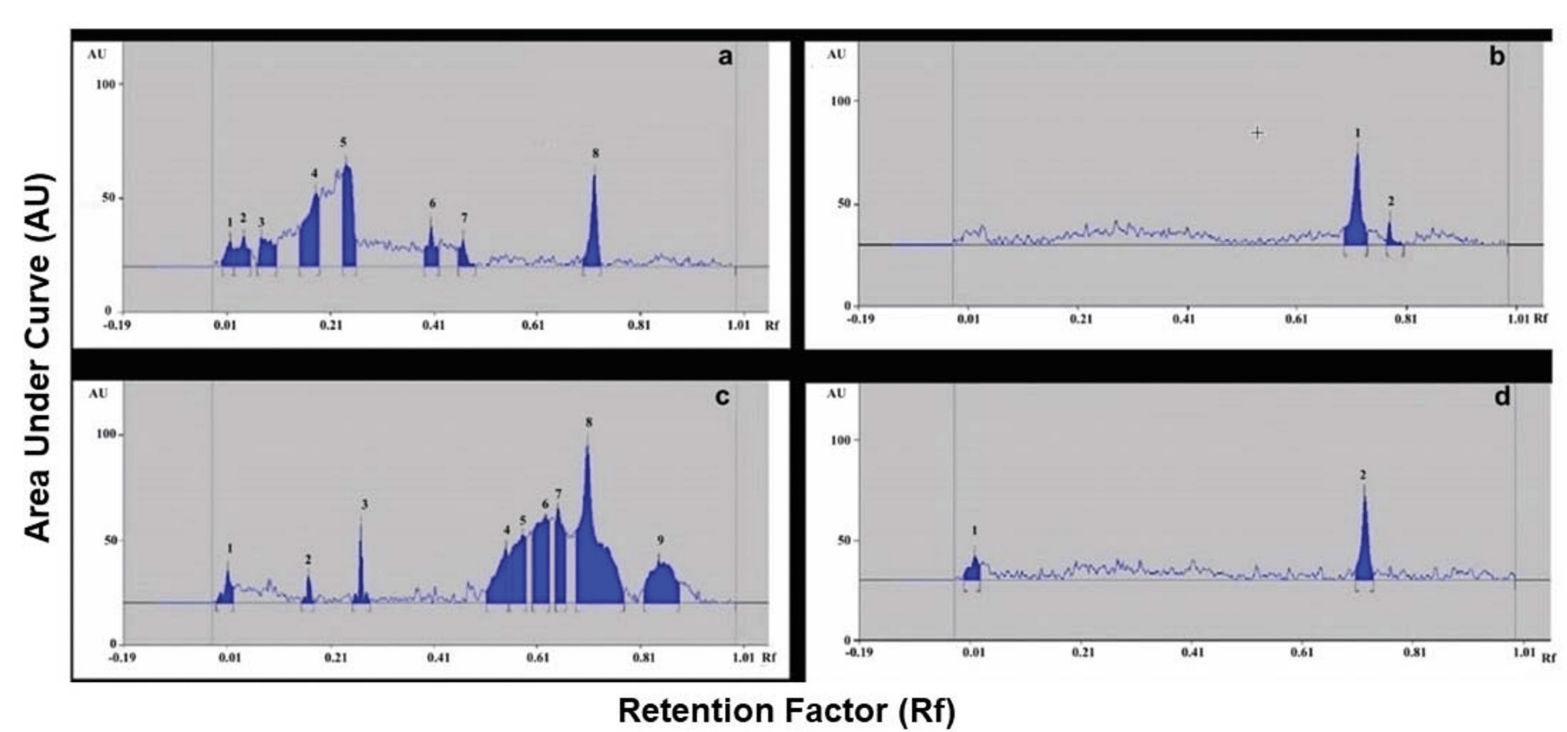
HPTLC chromatogram of organic solvent extracts of Commiphora caudata leaves and peak densitogram display at 400 nm a: n-Hexane extract, b: chloroform extract, c: ethanol extract, d: ethyl acetate extract HPTLC: high-performance thin-layer chromatography

An HPTLC analysis of all extracts revealed seven peaks in hexane extract, two peaks in chloroform extract, two peaks in ethyl acetate extract, and nine peaks with retention factors (Rf) varying from 0.01 to 0.85 in ethanol extract (Figure 1). Peak number 8 (Table 1) had an Rf value of 0.71, making it the most predominant compound in the ethanol extract.

**Table 1:** Rf value of different compounds from ethanol extract of Commiphora caudata leaves separated using the HPTLC method Rf: retention factor, HPTLC: high-performance thin-layer chromatography

Name of extract	Peak number	Rf value of peak (maximum Rf)	Area of peak	% area of peak
Ethanol extract	1	0.01	181.9	2.68
	2	0.17	91.0	1.34
	3	0.27	212.12	3.13
	4	0.55	554.8	8.18
	5	0.59	795.9	11.73
	6	0.63	1032.3	15.22
	7	0.66	691.8	10.20
	8	0.71	2380.3	35.09
	9	0.85	842.5	12.42

**Figure 2:**
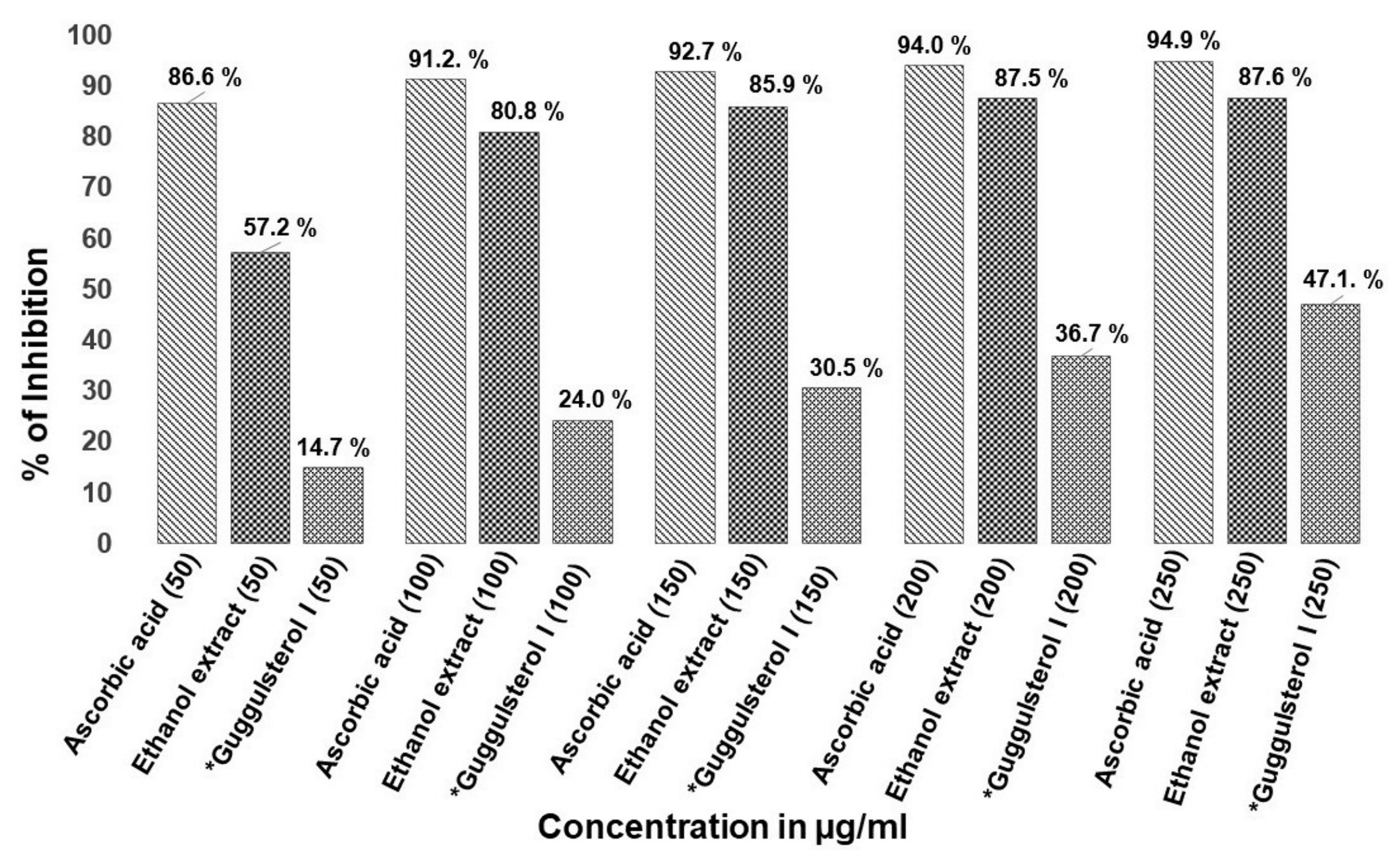
Radical scavenging activity of ethanol extract of Commiphora caudata leaves and isolated guggulsterol I using DPPH assay *Column purified compound DPPH: 2,2′-diphenyl-1-picrylhydrazyl

The DPPH assay confirmed the antioxidant activity of crude ethanol extract. The assay was performed with five different concentrations of ethanolic extract of 50, 100, 150, 200, and 250 µg/mL. At 250 µg/mL, the extract showed 87.69% inhibition compared to standard ascorbic acid (94.9%) at the same concentration, while the isolated compound had 47.1% of inhibition (Figure 2).

**Figure 3:**
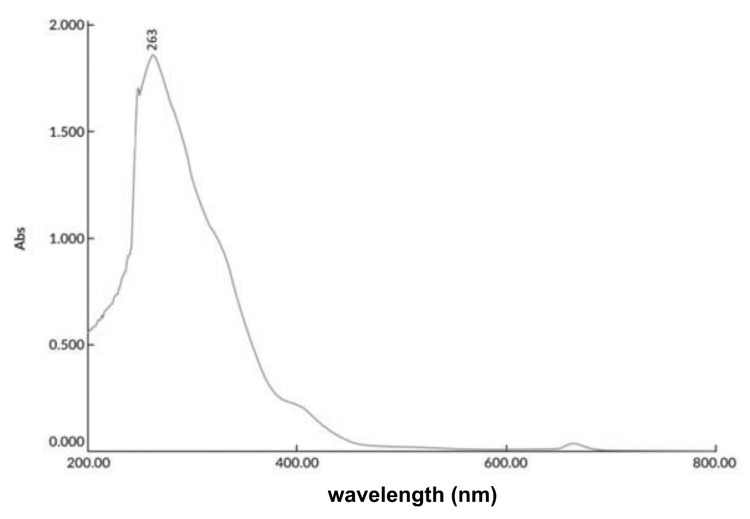
Absorption maxima of the isolated compound from column elution of ethanol extract of Commiphora caudata leaves

The column was eluted with n-hexane (100%), followed by a gradient increase of chloroform to 100%, and then a gradient increase of ethyl acetate to 100%. The collected fractions were analyzed on TLC plates using ethyl acetate-methanol-water (20:12:8) as the solvent system. Multiple spots were observed and confirmed by UV absorption. As the yield of concentrated fractions was less than 50 mg, further purification was not performed. The column was then eluted with ethyl acetate and ethanol up to 100%. Four fractions of 20 mL each were collected using a 25:75 ratio of ethyl acetate and ethanol. All four fractions produced a single spot on TLC with ethyl acetate-methanol-water (20:12:8) mobile phase. Analysis of these fractions with a UV spectrophotometer showed maximum absorption at 263 nm (Figure 3).

**Figure 4:**
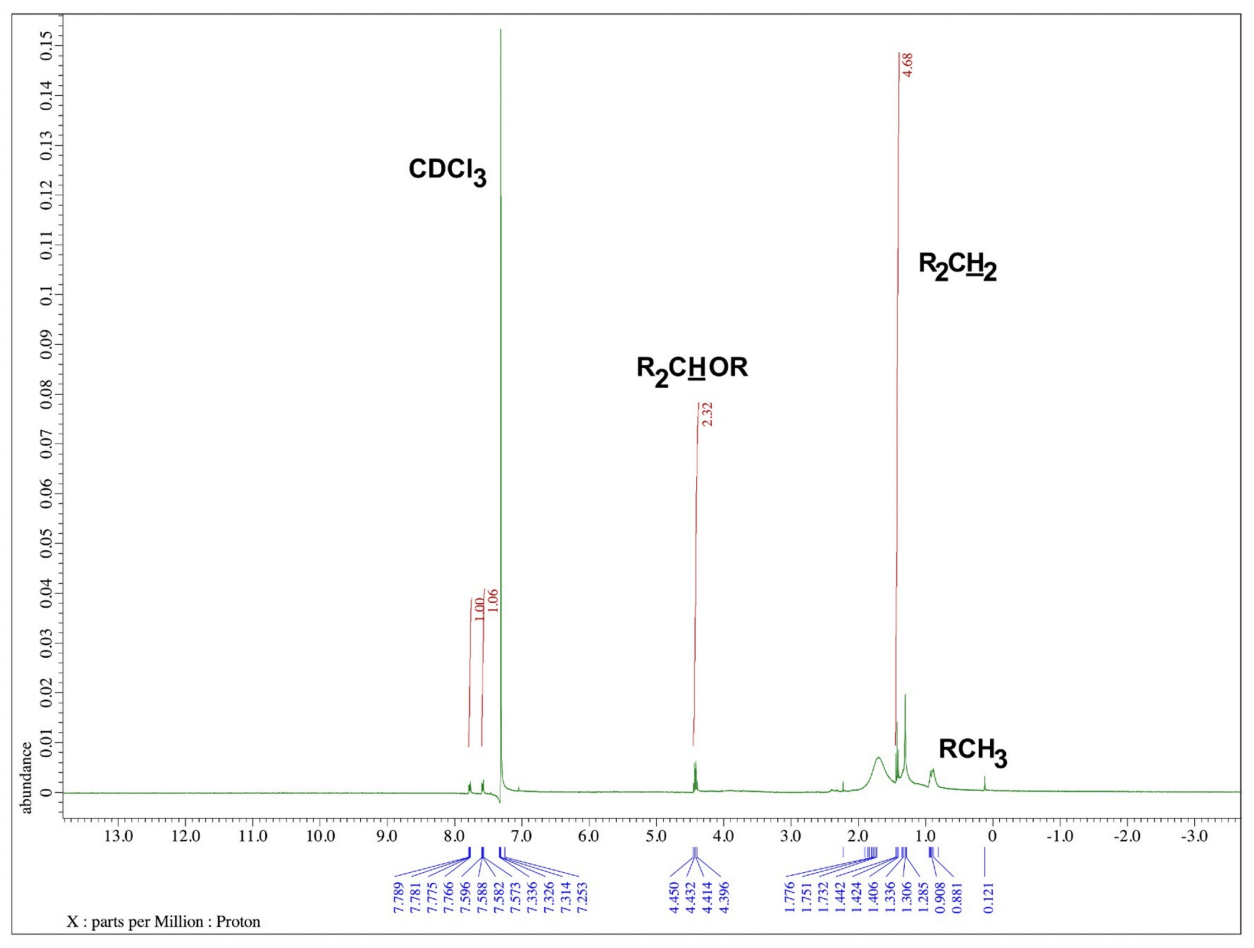
1H NMR in CDCl3 spectrum of the isolated compound from column elution of ethanol extract of Commiphora caudata leaves NMR: nuclear magnetic resonance

Based on this, all fractions were combined and concentrated for further analysis. The proton environment of the analyzed compound in 1H NMR in CDCl3 was identified as R2CH2 (1.28 ppm), RCH3 (0.92 ppm), and R2CHOR (4.3 ppm) (Figure 4).

**Figure 5:**
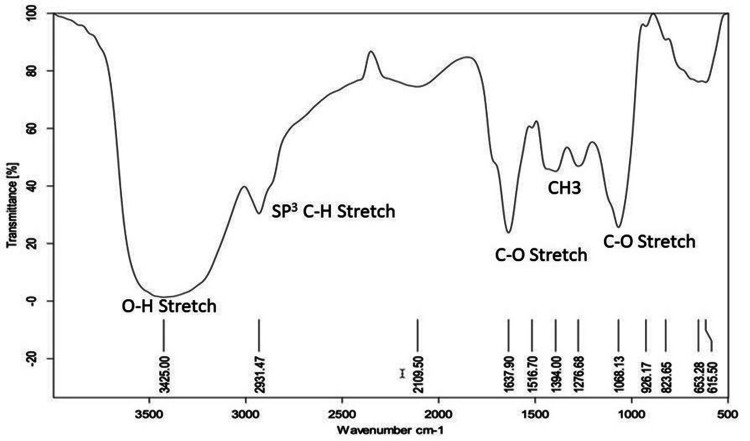
IR spectrum of the isolated compound from column elution of ethanol extract of Commiphora caudata leaves IR: infrared

IR spectral analysis showed an O-H stretch at 3425 cm^-1^, C-H (SP^3^) stretch at 2931, 1394, and 1276 cm^-1^, and a C-O stretch at 1637 and 1068 cm^-1^ (Figure 5).

**Figure 6:**
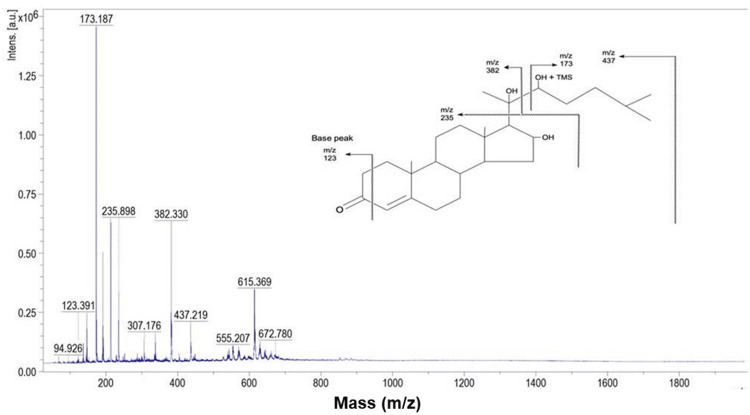
Mass spectroscopy analysis of the isolated compound from column elution of ethanol extract of Commiphora caudata leaves using Bruker UltrafleXtreme MALDI-TOF MALDI-TOF: matrix-assisted laser desorption/ionization-time of flight

Mass spectrometry analysis of this compound yielded m/z values of 437, 382, 307, 235, 173, 123, and 94.9 (Figure 6).

## Discussion

*Commiphora caudata* leaves are used in traditional and tribal medicine in Kerala, Tamil Nadu, and Karnataka, India, to treat painful inflammatory conditions. The leaves are one of the main ingredients in Amragandhi guggulu, described in the Ayurvedic pharmacopeia of India and recommended in liquid form for treating rheumatism, body aches, chafed or cracked soles, metabolic disorders, arthritis, and other conditions [18]. Crude extracts obtained from *C. caudata* leaves using various solvents were studied in detail under both in vitro and in vivo conditions and exhibited several biological activities, including antioxidant, hepatoprotective, analgesic, anti-inflammatory, antiarthritic, and antihyperlipidemic effects, as well as enhanced learning and memory [19]. Although these biological activities were proven for leaf extracts, the chemical constituents responsible for these activities were not completely established. The yield of phytoconstituents extracted was relatively lower than previous reports, but the preliminary qualitative chemical analysis was similar to that in other studies [9]. The ethanol extract contained gum resin, which showed antioxidant activity. Based on this, ethanol extract was selected for further investigation to isolate phytoconstituents.

Previous studies successfully used TLC to analyze plant extracts and identify phytoconstituents from cyclohexane, chloroform, ethyl acetate, methanolic, and aqueous extracts of* C. leptophloeos* bark, which contained phenolic compounds, flavonoids, and reducing sugars [20]. The HPTLC method is highly preferred for precisely detecting and quantifying phytoconstituents in plant extracts. This method was successfully used to estimate isomers of guggulsterone in blood serums, commercial formulations, and plant materials used in health supplements [21]. In this study, a predominant peak was detected in HPTLC with an Rf value of 0.71 and a percentage area of 37.1%. This fraction was further characterized to identify phytoconstituents present.

Free radical scavenging activity was tested with the crude ethanol extract, which showed 87.7% inhibition at a concentration of 250 µg/mL compared to standard ascorbic acid (94.9%) at the same concentration. However, the activity decreased to 47.1% with the isolated compound (Figure 2). The IC50 of the standard was found to be 175 µg/mL, while the test extract had an IC50 of 190 µg/mL, which was nine-fold higher than the value reported previously for this plant [22]. UV absorption λmax of the purified compound was very close to λmax guggulsterol reported from the *Commiphora* spp. at 257 nm [23]. Mass spectrum analysis of this compound gave an m/z value of 432. A compound with a similar molecular weight was identified as guggulsterol from the plant *C. mukul*, along with other functional moieties similar to the structure of guggulsterol I [24]. This was supported by the public domain molecular mass of guggulsterol I, which is 432.6 gm/mol (https://pubchem.ncbi.nlm.nih.gov/compound/Guggulsterol I). The mass intensities detected at m/z 382 (M-90), 307, and the parent ion at m/z 437 suggest a structure that might differ from cholesterol trimethylsilyl ether by adding 35 mass units, possibly due to elongation or side chain branching [25]. The mass spectrum of the trimethylsilyl derivative of 22R-OH cholesterol typically showed fragment ions at m/z 173 due to fission between C20 and C22 [26]. A similar fragment ion was detected in this study, with m/z 173 being the most abundant ion in the mass spectrum (Figure 6). The spectrum also revealed a cholesterol peak-like steroidal structure with an m/z value of 235, which had been previously reported with cholesteryl TMS ether [25].

Another hydroxyl group in the compound was likely attached to carbon 2 or 3, as the base peak was found at m/z 123. Based on the NMR and IR spectral evidence and by comparing the values with the literature, we hypothesized that the compound was guggulsterol I, with probable mass fragments as shown in Figure 6. A previous study showed that GC-MS analysis of organic solvent extracts of *Commiphora* stem bark identified 52 phytoconstituents, but guggulsterol or guggulsterone was not detected [14]. The bioassay-guided method successfully isolated guggulsterone from *C. gileadensis* and demonstrated its role as an antagonist of the bile acid receptor [27]. In this study, a bioassay-guided approach isolated guggulsterol I from the ethanol extract of *C. caudata *leaves. RSA with the chemical DPPH was performed at different concentration ranges from 50 to 250 µg/mL, showing a two- to four-fold decrease in the percentage of RSA compared with crude ethanol extract (Figure 2). This indicated that crude ethanol extract had higher RSA due to the presence of other compounds along with guggulsterol I. The RSA activity of purified guggulsterol I was concentration-dependent; at 50 µg/mL, it showed 14% RSA, while at 250 µg/mL, it showed 47.1% RSA compared to crude extract (Figure 2). This indicated that the crude ethanol extract had higher RSA due to the presence of other compounds along with guggulsterol I. This steroidal compound demonstrated good binding affinity with proteins that induce endoplasmic reticulum (ER) stress in docking studies [10], ameliorated inflammatory cytokines and oxidative stress markers, prevented ethidium bromide-induced apoptosis, and modulated neurotransmitter levels in a rat animal model [28].

The limitation of the present study was that a low quantity of guggulsterol I was isolated from the leaves of *C. caudata*, which was not sufficient to establish other biological activities reported in in silico studies. Only the ethanolic extract was column-purified and identified for antioxidant activity, but the phytoconstituents present in other organic extracts of *C. caudata* have yet to be established.

## Conclusions

The present study demonstrated that the ethanolic extract of *Commiphora caudata* leaves was detected with carbohydrates, proteins, steroids, and gum resin. In the in vitro conditions, it has been proven to possess antioxidant activity using DPPH. The bioassay-guided approach has been applied to ethanol extract to isolate the guggulsterol I compound, and this isolated compound exhibited antioxidant activity in the DPPH assay in a concentration-dependent manner. However, the antioxidant activity of the isolated compound was less than the crude ethanol extract and standard compound ascorbic acid. Based on the present study, we recommend a crude extract of *Commiphora caudata* leaves for better antioxidant activity rather than isolated compounds. It might be due to a low concentration of guggulsterol I and other phytoconstituent compounds present in the extract exhibiting synergistic activity.
